# Simultaneous gap arthroplasty and intraoral distraction and secondary contouring surgery for unilateral temporomandibular joint ankylosis

**DOI:** 10.1186/s40902-016-0058-0

**Published:** 2016-03-03

**Authors:** Aditi Sharma, Jun-Young Paeng, Tomohiro Yamada, Tae-Geon Kwon

**Affiliations:** 1 Department of Oral and Maxillofacial Surgery, School of Dentistry, Kyungpook National University, Daegu, 700-421 Korea; 2 Division of Maxillofacial Diagnostic and Surgical Sciences, Department of Dental Science, Faculty of Dental Science, Kyushu University, Fukuoka, Japan

**Keywords:** Temporomandibular joint, Ankylosis, Intraoral distraction, Gap arthroplasty, Asymmetry

## Abstract

**Background:**

Temporomandibular joint (TMJ) ankylosis can be accompanied by various degrees of functional and esthetic problems. Adequate mouth opening, occlusal stability, and harmonious facial form are the main goals of treatment for ankylosis. Distraction osteogenesis has proven to be an excellent treatment for lengthening the ramus-condyle unit. However, various timings for distraction have been suggested, and there is no consensus on selection criteria for performing the procedure in stages or simultaneously with other treatments.

**Case presentation:**

In this case report, concomitant intraoral distraction and gap arthroplasty was planned to treat TMJ ankylosis and associated facial asymmetry. After gap arthroplasty and 23 mm of distraction, the ramus-condyle segment was successfully lengthened and mouth opening range was significantly increased. The resultant interocclusal space was stably maintained with an occlusal splint for 4 months after distraction. Finally, good occlusion was achieved after prosthetic treatment. The remaining mandibular asymmetry was corrected with osseous contouring and augmentation surgery. The mouth-opening range was maintained at 35 mm 24 months after treatment.

**Conclusion:**

Gap arthroplasty with intraoral distraction as a one-stage treatment and subsequent contouring surgery can be applied to correct ankylosis with moderate malocclusion and facial asymmetry.

## Background

Temporomandibular joint (TMJ) ankylosis is a fusion of the mandibular condyle to the base of the skull, which causes problems in speech, mastication, and facial appearance, and sleep-disordered breathing. The main concept of ankylosis treatment includes complete resection of the ankylotic block, creation of a new joint lining with an interpositional substance, and reconstruction of the skeletal deformity. This protocol was fully demonstrated by Kaban et al. [1]. Costochondral grafts—which have the advantage of being an autogenous material with a cartilaginous articulating surface, allowing potential for growth and adaption—are commonly used to reconstruct the mandibular condyle. However, recent analysis has shown that gap arthroplasty produces better postoperative maximal mouth opening than does costochondral graft [2]. Even after successful release of ankylosis, mandibular deformity may become aggravated after surgery because of the loss of ramus-condyle height. Total joint reconstruction or distraction osteogenesis has therefore been suggested to reconstruct the ramus-condyle unit [3, 4].

Recently, distraction osteogenesis has proven to be an ideal treatment for TMJ ankylosis [5, 6]. However, the best timing for the distraction is still controversial. Some authors have suggested that distraction should be performed after gap arthroplasty [7–9], while others have performed distraction first, followed by gap arthroplasty at the time of distractor removal [10, 11]. Simultaneous gap arthroplasty and distraction osteogenesis for treatment of micrognathia in TMJ ankylosis has also been reported [12–15]. Recently, Zhu et al. [16] recommended the staged approach in cases of severe malocclusion and deformity, as it is difficult to establish occlusion and facial harmony in a one-stage treatment. In cases with mild-to-moderate malocclusion, dental compensation with prosthetic or orthodontic treatment can be performed after simultaneous removal of the ankylotic mass and skeletal deformities. However, the technical details of simultaneous gap arthroplasty and distraction had not yet been fully documented, especially in terms of establishing occlusion during the treatment process.

In the reported case, the authors performed gap arthroplasty and intraoral distraction as a one-stage treatment, followed by mandibular contouring surgery and prosthetic treatment for unilateral TMJ ankylosis with facial asymmetry. The results demonstrate that stable outcomes after concomitant gap arthroplasty and distraction can be ensured by maintaining the occlusion with a splint until prosthetic treatment.

## Case presentation

**Fig. 1:**
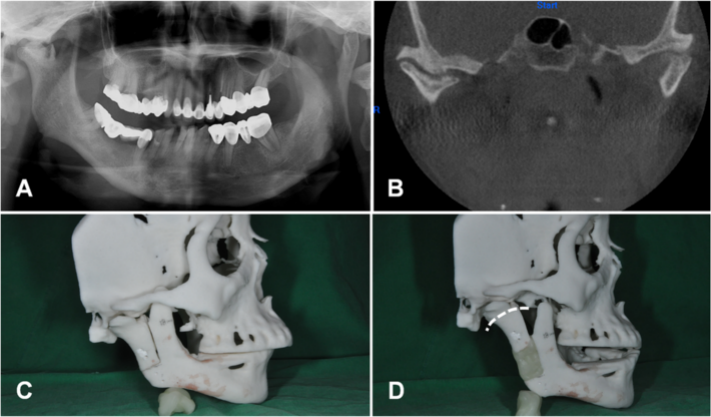
**a** The panoramic radiograph showed right TMJ ankylosis and facial asymmetry. **b** Coronal CT image shows fibrous adhesion around the right TMJ. **c** Reverse L-shape osteotomy line for distraction osteogenesis. **d** Distractor applied on rapid prototype model, and proposed line of osteotomy (*white dotted line*) for ankylosis release. A 23-mm distraction of the condyle-ramus segment was planned

**Fig. 2:**
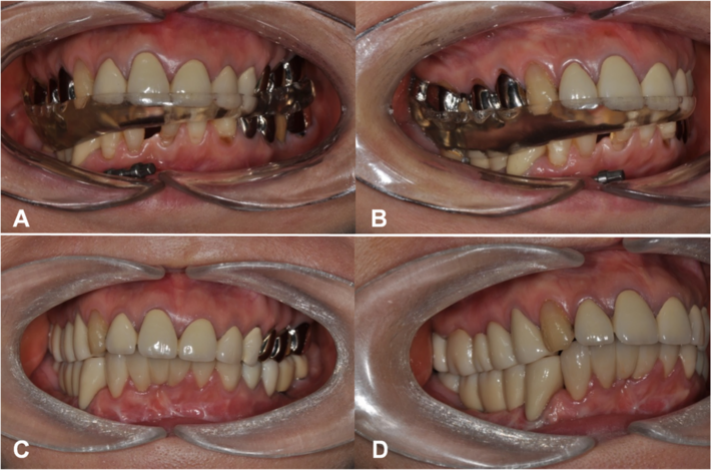
Intraoral photographs. **a**, **b** Occlusal changes after distraction. Posterior lateral open bite was maintained for 4 months during the consolidation period, until the final prosthetic treatment. **c**, **d** The occlusal splint was removed, and the remaining lateral open bite was closed by final prosthetic treatment

A 47-year-old woman presented with a complaint of facial asymmetry and limitation of mouth opening. The patient had a history of untreated chin trauma in adolescence. Clinical examination showed maxillary canting and deviation of the mandibular dental midline and chin to the right. Maximal mouth opening was limited to 23 mm. Radiological examination showed fibrous ankylosis with elongated coronoid process and shortened ramal height on the right side (Fig. 1a, b). A reverse L-shape osteotomy line was marked on the three-dimensional rapid prototype model (Fig. 1c). Release of the ankylotic block by 10 mm was required (white line). To achieve symmetrical ramal height, a total of 20 mm of ramal lengthening was planned (Fig. 1d). Under general anesthesia, the ankylotic joint was exposed via a preauricular approach. Gap arthroplasty was performed, leaving a 10-mm space between the glenoid fossa and mandible. The elongated ipsilateral coronoid process was resected. Mouth opening of 40 mm was achieved. A reverse L-shaped osteotomy line was made on the outer cortex of the ramal bone via a preauricular and intraoral approach. A 25-mm Ramus distractor (KLS Martin, Tuttlingen, Germany) was adapted to the ramus, and predrilling was performed on each side of the horizontal osteotomy line by using the angled screw driver, Angulus (KLS Martin, Tuttlingen, Germany). The designed osteotomy was created while maintaining the soft tissue attachment of the medial side of the proximal ramal segment. Finally, the distractor was fixed with miniscrews and its function was tested. After a 7-day latency period, the mandible was distracted to 23 mm at a rate of 0.5 mm × 2 times per day. During the distraction period, the patient performed mouth-opening exercises composed of voluntary, gentle mouth opening for 5 cycles (5 times/cycle) per day. After the distraction period, lateral open bite was noted on the right side. The ledge of the distraction device was removed. To maintain and stabilize the occlusion, a resin splint was applied for 4 months during the consolidation period until removal of the distractor (Fig. 2a, b). The occlusal splint was removed, and the remaining lateral open bite was closed by prosthetic treatment (Fig. 2c, d).

**Fig. 3:**
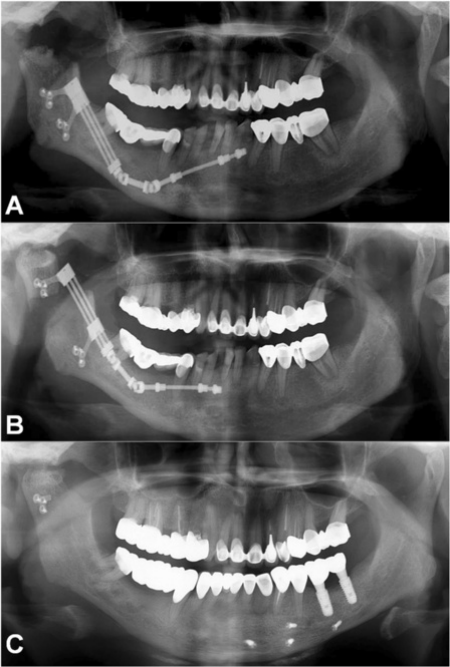
Comparison of pre- and postdistraction panoramic views. **a** Immediately after simultaneous gap arthroplasty and start of distraction osteogenesis. **b** After completion of a 23-day distraction period at a rate of 0.5 mm, two times per day). **c** After a 16-month follow-up period

**Fig. 4:**
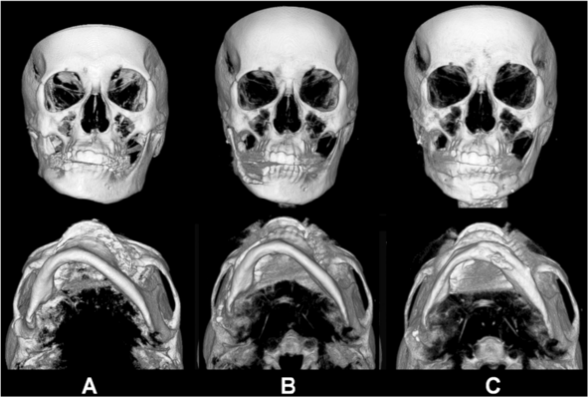
Three-dimensional computed tomography images taken at each successive surgical step. **a** Preoperative. **b** After gap arthroplasty and distraction osteogenesis. **c** After mandibular contouring surgery to correct residual asymmetry of the mandible

**Fig. 5:**
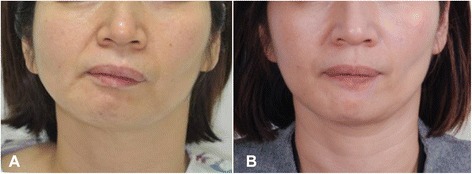
Facial photographs before **(a)** and after **(b)** treatment. The patient exhibits significantly improved symmetry and stable skeletal position after treatment

At the time of distractor removal, residual mandibular asymmetry was corrected by decortication and shaving of the right mandibular body, and augmentation of the left mandibular body using the decorticated bone from the contralateral side. After a 24-month follow-up period, mouth opening was maintained at 35 mm. Comparison of pre- and postdistraction panoramic views showed a remarkable ramal height increase (Fig. 3). Three-dimensional computed tomography images taken at each successive surgical step are shown in Fig. 4. The patient exhibited significantly improved symmetry and stable skeletal position after treatment (Fig. 5).

### Discussion

There are several fundamental elements for successful treatment for TMJ ankylosis and related dentofacial deformity: establishment of adequate mouth-opening range, complete removal of the ankylotic block to prevent reankylosis, and establishment of balanced facial appearance after surgery. To achieve these goals, various approaches have been suggested. After the introduction of distraction osteogenesis for mandibular ramus lengthening, some surgeons attempted simultaneous correction of all deformities by performing distraction at the time of ankylotic mass removal. Critics of this approach have suggested that after release of the ankylotic block, changes in the mandibular position cannot be completely controlled during the distraction period. During distraction, the condylar segment can move toward the condylar fossa and become positioned closer to the articular surface, which can result in reankylosis if adequate physical therapy is not applied. However, it is not easy to perform active physical therapy during the distraction period because of postsurgical pain or discomfort. For these reasons, a staged operation for TMJ ankylosis—comprising ankylosis release as the first surgery, and distraction as the second—has been proposed [7–9]. However, after reports of two successful cases of simultaneous gap arthroplasty and distraction [12, 13] in 1999, many authors subsequently reported similar successes [14, 15, 17]. Other surgeons have chosen to apply distraction as the first-stage surgery, followed by gap arthroplasty [10, 11]. As the distraction in such cases is applied to an ankylosed joint without mobility, it is easy to push the mandible forward during distraction. However, performing minimal arthroplasty without vertical bony resection in the distracted ramus-condyle unit is not easy, and carries the risk of reankylosis.

Zhu et al. [16] proposed that one-stage surgical treatment is indicated for patients with mild-to-moderate preoperative malocclusion and skeletal deformities. Staged treatment, on the other hand, is better suited to achieve a more stable postsurgical outcome in patients with severe dentofacial deformities. This is in accordance with our previous report showing the advantages of staged treatment; it promotes early postoperative engagement in active mouth-opening exercise, allows sufficient time to monitor malocclusion, and may reduce the chances of reankylosis [9]. The current patient had unilateral fibrous ankylosis on the right side (Sawhney’s classification [18] Type I) and showed mild occlusion and facial asymmetry that could be corrected by single-stage surgery. On the basis of our previous report [9], the current case, and remarks from another report [16], we believe that the timing of distraction (i.e., after or simultaneously with the gap arthroplasty) can be determined by assessing the possibility of occlusal correction by postoperative orthodontic or prosthetic treatment to prevent postdistraction occlusal change.

It has been recommended that viability of the osteotomized segment can be maintained by preserving the intact medial pterygoid and masseter muscle during the distraction period [17], which is in accordance with our technique. During distraction of the ramus-condyle segment, direct bone-to-bone contact should be avoided, especially in the case of bony ankylosis. Therefore, it is advisable to use interpositional grafts, which can serve as a physical barrier to reduce pressure on the reconstructed condyle. In our case report, an interpositional graft was not used because the ankylosis was of the fibrous type.

Yoon et al. [19] reported simultaneous distraction and gap arthroplasty using an intraoral distractor via an extraoral approach. Xu et al. [17] reported use of a single preauricular incision for gap arthroplasty and distraction, with a penetrating distraction port at the preauricular incision. We positioned an intraoral distractor for lengthening the ramus-condyle unit via an intraoral approach, and used a preauricular incision for ankylosis release to minimize patient morbidity and extraoral scarring.

With this case report, we suggest that fibrous ankylosis with mild-to-moderate malocclusion and facial asymmetry can be successfully treated by simultaneous gap arthroplasty and distraction as a single-stage treatment. Residual skeletal asymmetry can subsequently be corrected by secondary contouring surgery. Occlusal stability plays a major role in promoting a favorable treatment outcome. In the present case, the definite occlusal instability after the distraction period was effectively managed by subsequent occlusal splint application and prosthetic treatment. If lateral and posterior open bite after distraction of the ramus-condyle unit cannot be managed by occlusal modifications such as splint application and subsequent orthodontic or prosthodontic treatment, adequate condyle-fossa relation and facial symmetry cannot be maintained. We therefore believe that in cases with severe joint ankylosis requiring complete removal of the condyle head and neck to the level of the coronoid notch, a staged operation is advantageous compared with single-stage distraction accompanied by gap arthroplasty [9].

## Conclusions

In this case report, we experienced a favorable outcome without evident relapse after 24 months of follow-up. Therefore, we suggest that single- or multistage treatment can be applied depending on the degree of malocclusion or occlusal instability, which is a reflection of TMJ ankylosis severity. Gap arthroplasty with intraoral distraction as a one-stage treatment and subsequent contouring surgery can be applied to correct ankylosis with moderate malocclusion and facial asymmetry.

## Consent

Written informed consent was obtained from the patient for the publication of this report and any accompanying images.

## References

[1] Kaban LB, Bouchard C, Troulis MJ (2009). A protocol for management of temporomandibular joint ankylosis in children. J Oral Maxillofac Surg.

[2] Katsnelson A, Markiewicz MR, Keith DA, Dodson TB (2012). Operative management of temporomandibular joint ankylosis: a systematic review and meta-analysis. J Oral Maxillofac Surg.

[3] Loveless TP, Bjornland T, Dodson TB, Keith DA (2010). Efficacy of temporomandibular joint ankylosis surgical treatment. J Oral Maxillofac Surg.

[4] Feiyun P, Wei L, Jun C, Xin X, Zhuojin S, Fengguo Y (2010). Simultaneous correction of bilateral temporomandibular joint ankylosis with mandibular micrognathia using internal distraction osteogenesis and 3-dimensional craniomaxillofacial models. J Oral Maxillofac Surg..

[5] Schwartz HC, Relle RJ (2008). Distraction osteogenesis for temporomandibular joint reconstruction. J Oral Maxillofac Surg.

[6] Bansal V, Singh S, Garg N, Dubey P (2014). Transport distraction osteogenesis as a method of reconstruction of the temporomandibular joint following gap arthroplasty for post-traumatic ankylosis in children: a clinical and radiological prospective assessment of outcome. Int J Oral Maxillofac Surg.

[7] Hegab AF (2015). Outcome of surgical protocol for treatment of temporomandibular joint ankylosis based on the pathogenesis of ankylosis and re-ankylosis. A prospective clinical study of 14 patients. J Oral Maxillofac Surg.

[8] Lopez EN, Dogliotti PL (2004). Treatment of temporomandibular joint ankylosis in children: is it necessary to perform mandibular distraction simultaneously?. J Craniofac Surg.

[9] Kwon TG, Park HS, Kim JB, Shin HI (2006). Staged surgical treatment for temporomandibular joint ankylosis: intraoral distraction after temporalis muscle flap reconstruction. J Oral Maxillofac Surg.

[10] Sadakah AA, Elgazzar RF, Abdelhady AI (2006). Intraoral distraction osteogenesis for the correction of facial deformities following temporomandibular joint ankylosis: a modified technique. Int J Oral Maxillofac Surg.

[11] Shang H, Xue Y, Liu Y, Zhao J, He L (2012). Modified internal mandibular distraction osteogenesis in the treatment of micrognathia secondary to temporomandibular joint ankylosis: 4-year follow-up of a case. J Craniomaxillofac Surg.

[12] Dean A, Alamillos F (1999). Mandibular distraction in temporomandibular joint ankylosis. Plast Reconstr Surg.

[13] Papageorge MB, Apostolidis C (1999). Simultaneous mandibular distraction and arthroplasty in a patient with temporomandibular joint ankylosis and mandibular hypoplasia. J Oral Maxillofac Surg.

[14] Yu H, Shen G, Zhang S, Wang X (2009). Gap arthroplasty combined with distraction osteogenesis in the treatment of unilateral ankylosis of the temporomandibular joint and micrognathia. Br J Oral Maxillofac Surg.

[15] Cascone P, Agrillo A, Spuntarelli G, Arangio P, Iannetti G (2002). Combined surgical therapy of temporomandibular joint ankylosis and secondary deformity using intraoral distraction. J Craniofac Surg.

[16] Zhu S, Wang D, Yin Q, Hu J (2013). Treatment guidelines for temporomandibular joint ankylosis with secondary dentofacial deformities in adults. J Craniomaxillofac Surg.

[17] Xu J, Long X, Cheng AH, Cai H, Deng M, Meng Q (2015). Modified condylar distraction osteogenesis via single preauricular incision for treatment of temporomandibular joint ankylosis. J Craniofac Surg.

[18] Sawhney CP (1986). Bony ankylosis of the temporomandibular joint: follow-up of 70 patients treated with arthroplasty and acrylic spacer interposition. Plast Reconstr Surg.

[19] Yoon HJ, Kim HG (2002). Intraoral mandibular distraction osteogenesis in facial asymmetry patients with unilateral temporomandibular joint bony ankylosis. Int J Oral Maxillofac Surg.

